# Supplementary material for: REV1 coordinates a multi-faceted tolerance response to DNA alkylation damage and prevents chromosome shattering in Drosophila melanogaster
Source: PLoS Genet. 2024 Jul 29;20(7):e1011181. doi: 10.1371/journal.pgen.1011181 (PMC11309488; doi:10.1371/journal.pgen.1011181)
Supplement: S1 Fig — (A) The rev1Δ mutant was created through imprecise excision of a P transposable element inserted in the 5’ UTR of REV1. The resulting deletion removes 4531 bp downstream of the P element (the entire coding sequence of the REV1 gene), while retaining 951 bp of the P element 5’ end. White regions inidicate the protein coding region, while shaded regions indicate the untranslated regions. Numbers indicate nulceotide position from the start of transcription. (B) The spn-A057 allele is a V205A missense mutation that has been shown to act as a null allele [107]. The spn-A093 allele is a Q70stop nonsense mutation that also behaves as a null allele [107]. Both mutations were created through EMS mutagenesis. Mutation positions are indicated with vertical lines. Numbers indicate amino acid positions. (C) The brca2KO allele was created through ends-out homologous recombination and replaces the entire coding sequence of BRCA2 with the mini-white gene [108]. (D) The pol eta12 and mus2053B (rev33B) mutants were described in [59]. These mutations were created through imprecise excision of a P element. Both mutations are large deletions that result in frameshifts and premature stop codons and are predicted to create null alleles. (PDF) [file pgen.1011181.s001.pdf]

Supplementary Figure 1
Khodaverdian *et al.*

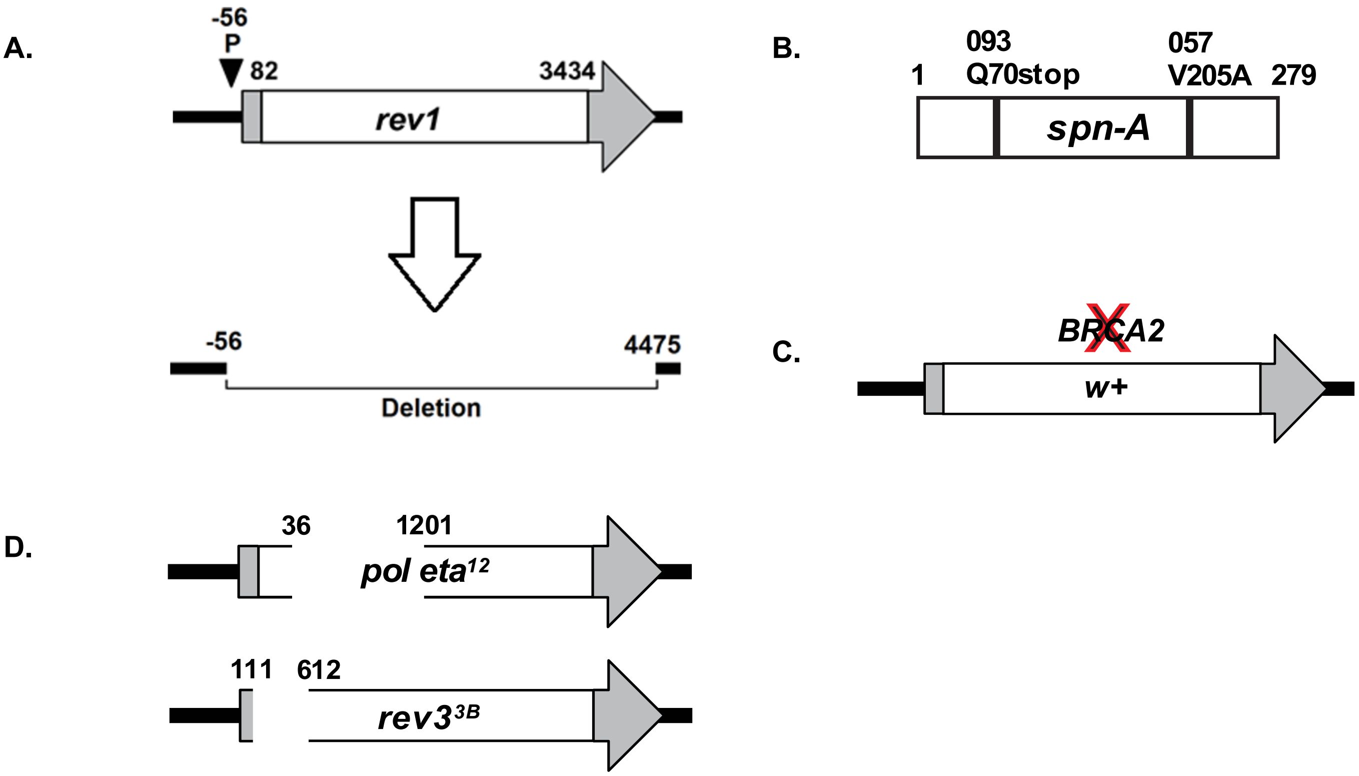

S1 Fig: Mutants used in this study.

(A) The *rev1*Δ mutant was created through imprecise excision of a *P* transposable element inserted in the 5' UTR of *REV1*. The resulting deletion removes 4531 bp downstream of the *P* element (the entire coding sequence of the *REV1* gene), while retaining 951 bp of the *P* element 5' end. White regions inidicate the protein coding region, while shaded regions indicate the untranslated regions. Numbers indicate nulceotide position from the start of transcription.

(B) The *spn-A*^{057} allele is a V205A missense mutation that has been shown to act as a null allele [1]. The *spn-A*^{093} allele is a Q70stop nonsense mutation that also behaves as a null allele [1]. Both mutations were created through EMS mutagenesis. Mutation positions are indicated with vertical lines. Numbers indicate amino acid positions.

(C) The *brca2*^{KO} allele was created through ends-out homologous recombination and replaces the entire coding sequence of *BRCA2* with the *mini-white* gene [2].

(D) The *pol eta*^{12} and *mus205*^{3B} (*rev3*^{3B}) mutants were described in [3]. These mutations were created through imprecise excision of a *P* element. Both mutations are large deletions that result in frameshifts and premature stop codons and are predicted to create null alleles.

Supplementary Figure 1
Khodaverdian *et al.*

References

1. Staeva-Vieira E, Yoo S, Lehmann R. An essential role of DmRad51/SpnA in DNA repair and meiotic checkpoint control. EMBO J. 2003;22(21):5863-74. doi: 10.1093/emboj/cdg564. PubMed PMID: 14592983; PubMed Central PMCID: PMCPMC275421.
2. Klovstad M, Abdu U, Schupbach T. Drosophila brca2 is required for mitotic and meiotic DNA repair and efficient activation of the meiotic recombination checkpoint. PLoS Genet. 2008;4(2):e31. doi: 10.1371/journal.pgen.0040031. PubMed PMID: 18266476; PubMed Central PMCID: PMCPMC2233675.
3. Kane DP, Shusterman M, Rong Y, McVey M. Competition between replicative and translesion polymerases during homologous recombination repair in Drosophila. PLoS Genet. 2012;8(4):e1002659. doi: 10.1371/journal.pgen.1002659. PubMed PMID: 22532806; PubMed Central PMCID: PMCPMC3330096.
