# Supplementary material for: REV1 coordinates a multi-faceted tolerance response to DNA alkylation damage and prevents chromosome shattering in Drosophila melanogaster
Source: PLoS Genet. 2024 Jul 29;20(7):e1011181. doi: 10.1371/journal.pgen.1011181 (PMC11309488; doi:10.1371/journal.pgen.1011181)
Supplement: S2 Fig — (A) Cas9-expressing S2R+ cells [66] were transfected with pLib6.4 containing a sgRNA targeting a sequence in the first exon of REV1 and the cells were passaged for 30 days under puromycin selection. Genomic DNA was extracted from the transfected cells, PCR was used to amplify the region flanking the Cas9 cut site, the PCR product was Sanger sequenced, and knockout efficiency was analyzed using ICE analysis (Synthego). The inferred sequences present in the edited population and their relative proportions are indicated in the contribution column. The cut site is represented by a black vertical dotted line. By ICE analysis, 95% of the cells possess indels and 83% of the cells have indels that are predicted to create null mutations in REV1. (B) The cell population analyzed in (A) was treated with 0.004% MMS (v/v) for 4 days. Cells were washed and grown in fresh media for 1 day, after which genomic DNA was isolated and subjected to ICE analysis as above. Post MMS treatment, 86% of the cells have indel mutations, of which 71% are predicted to create null mutations. (PDF) [file pgen.1011181.s002.pdf]

Supplementary Figure 2
Khodaverdian *et al.*

A. S2R+ cells pre-MMS treatment

sgRNA target: AGCCAAGAAATCTAACTTG
PAM sequence: AGG
Indels: 95%
Knockout score: 83

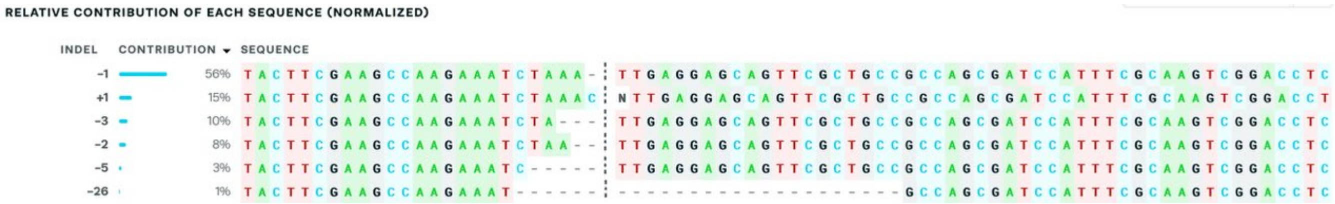

B. S2R+ cells post-MMS treatment

sgRNA guide target: AGCCAAGAAATCTAAACTTG
PAM sequence: AGG
Indels: 85%
Knockout score: 71

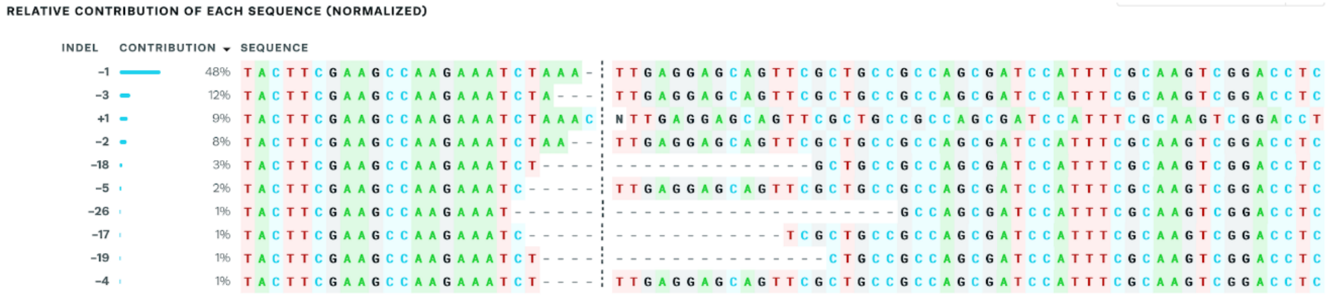

S2 Fig (extension to Fig 1C): The frequency of *rev1* mutant S2 cells does not change after treatment with MMS.

(A) Cas9-expressing S2R+ cells [1] were transfected with pLib6.4 containing a sgRNA targeting a sequence in the first exon of REV1 and the cells were passaged for 30 days under puromycin selection. Genomic DNA was extracted from the transfected cells, PCR was used to amplify the region flanking the Cas9 cut site, the PCR product was Sanger sequenced, and knockout efficiency was analyzed using ICE analysis (Synthego). The inferred sequences present in the edited population and their relative proportions are indicated in the contribution column. The cut site is represented by a black vertical dotted line. By ICE analysis, 95% of the cells possess indels and 83% of the cells have indels that are predicted to create null mutations in REV1. Synthego Performance Analysis, ICE Analysis. 2019. v3.0. Synthego; [5-5-24].

(B) The cell population analyzed in (A) was treated with 0.004% MMS (v/v) for 4 days. Cells were washed and grown in fresh media for 1 day, after which genomic DNA was isolated and subjected to ICE analysis as above. Post MMS treatment, 86% of the cells have indel mutations, of which 71% are predicted to create null mutations.

Supplementary Figure 2
Khodaverdian *et al.*

References

1. Viswanatha R, Li Z, Hu Y, Perrimon N. Pooled genome-wide CRISPR screening for basal and context-specific fitness gene essentiality in Drosophila cells. Elife. 2018;7. Epub 20180727. doi: 10.7554/eLife.36333. PubMed PMID: 30051818; PubMed Central PMCID: PMCPMC6063728.
