# Supplementary material for: REV1 coordinates a multi-faceted tolerance response to DNA alkylation damage and prevents chromosome shattering in Drosophila melanogaster
Source: PLoS Genet. 2024 Jul 29;20(7):e1011181. doi: 10.1371/journal.pgen.1011181 (PMC11309488; doi:10.1371/journal.pgen.1011181)
Supplement: S3 Fig — Brains were dissected from rev1 mutant third-instar larvae and squashed according to [112]. Representative images of catastrophic events involving multiple breaks, chromosome shattering, and aneuploidy are shown. (PDF) [file pgen.1011181.s003.pdf]

Supplementary Figure 3
Khodaverdian *et al.*

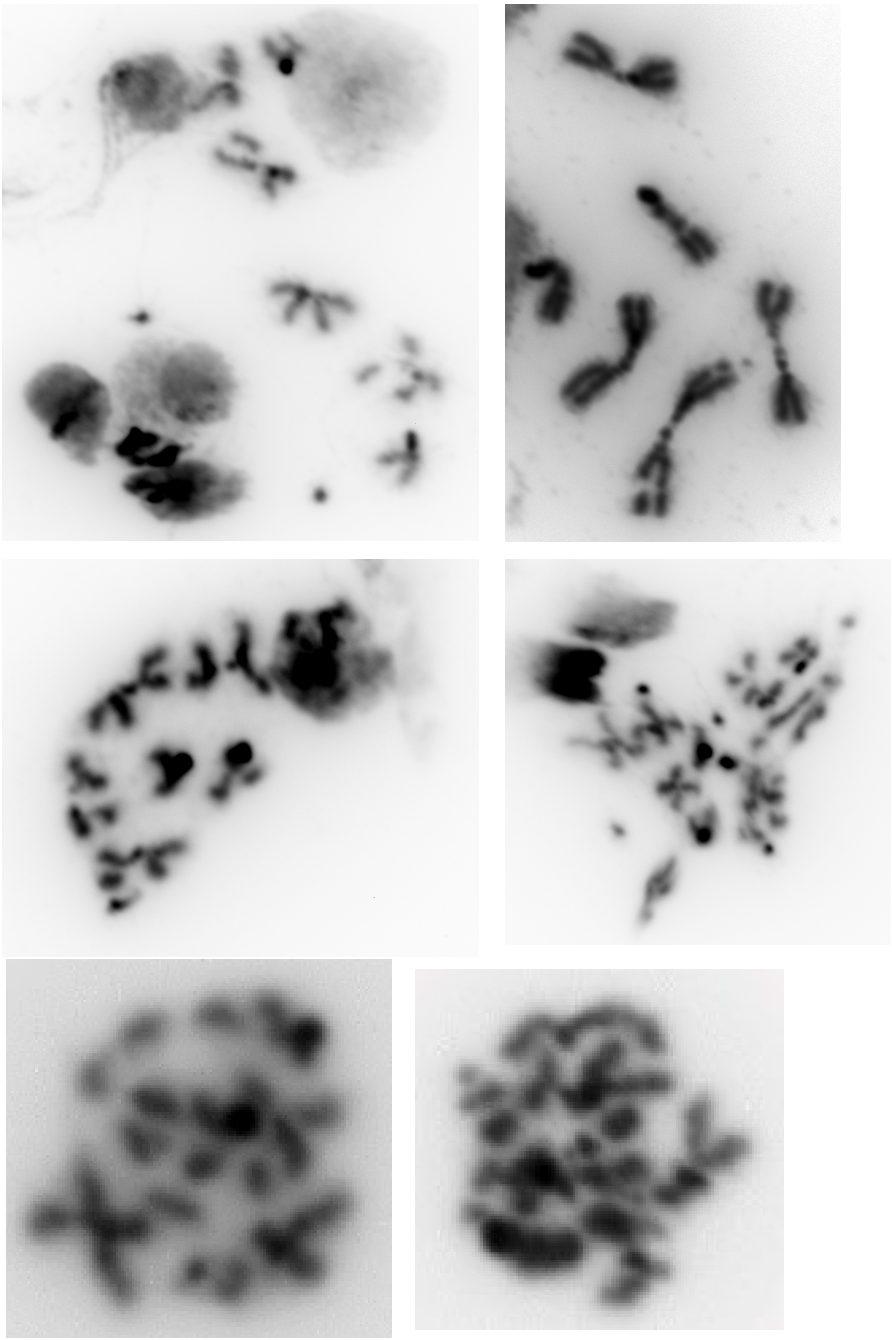

S3 Fig (extension to Fig 3): Examples of aberrant *rev1Δ* mitotic spreads.

Brains were dissected from *rev1* mutant third-instar larvae and squashed according to [1]. Representative images of catastrophic events involving multiple breaks, chromosome shattering, and aneuploidy are shown.

Supplementary Figure 3
Khodaverdian *et al.*

References

1. Gatti M, Santini G, Pimpinelli S, Olivieri G. Lack of spontaneous sister chromatid exchanges in somatic cells of Drosophila melanogaster. Genetics. 1979;91(2):255-74. doi: 10.1093/genetics/91.2.255. PubMed PMID: 109350; PubMed Central PMCID: PMCPMC1216365.
