# Supplementary material for: REV1 coordinates a multi-faceted tolerance response to DNA alkylation damage and prevents chromosome shattering in Drosophila melanogaster
Source: PLoS Genet. 2024 Jul 29;20(7):e1011181. doi: 10.1371/journal.pgen.1011181 (PMC11309488; doi:10.1371/journal.pgen.1011181)
Supplement: S4 Fig — Translesion synthesis (TLS) is favored in rapidly dividing cells in imaginal discs and neuroblasts, as indicated by the darker arrows. REV1 may recruit multiple TLS polymerases to the fork for immediate bypass during replication. Alternatively, PCNA-Ub may recruit Pol η and Pol ζ to single-strand gaps behind the fork to carry out post-replication repair. When TLS is compromised, either in REVΔCTD flies or flies lacking one or more TLS polymerases, RAD51-mediated fork regression or template switching can compensate. (PDF) [file pgen.1011181.s004.pdf]

Supplementary Figure 4
Khodaverdian *et al.*

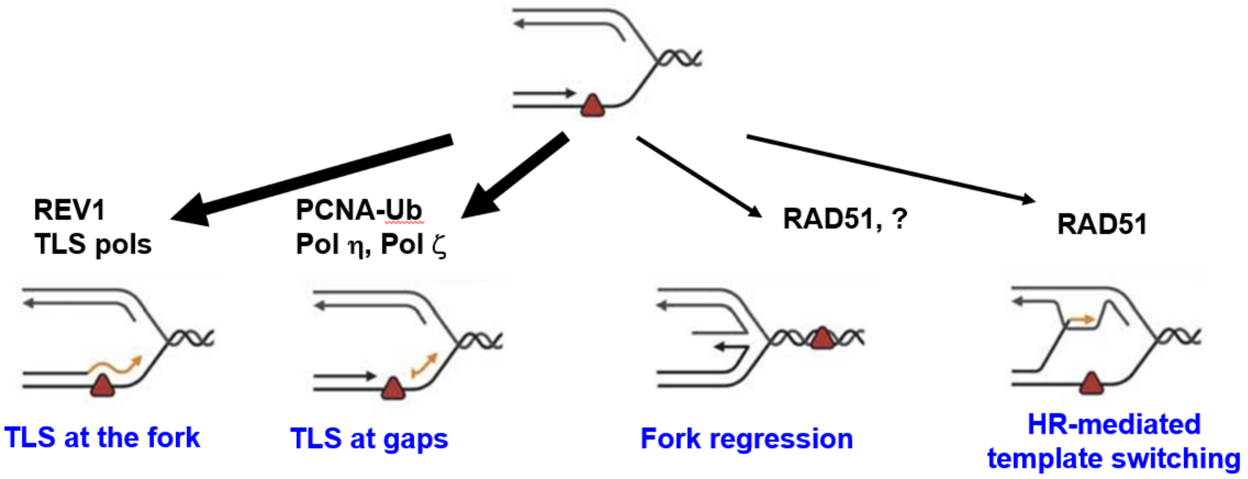

S4 Fig: A model for DNA damage tolerance preferences in Drosophila.

Translesion synthesis (TLS) is favored in rapidly dividing cells in imaginal discs and neuroblasts, as indicated by the darker arrows. REV1 may recruit multiple TLS polymerases to the fork for immediate bypass during replication. Alternatively, PCNA-Ub may recruit Pol η and Pol ζ to single-strand gaps behind the fork to carry out post-replication repair. When TLS is compromised, either in *REV∆CTD* flies or flies lacking one or more TLS polymerases, RAD51-mediated fork regression or template switching can compensate.
